# Serum Sphingolipid Variations Associate with Hepatic Decompensation and Survival in Patients with Cirrhosis

**DOI:** 10.1371/journal.pone.0138130

**Published:** 2015-09-18

**Authors:** Georgios Grammatikos, Nerea Ferreiròs, Oliver Waidmann, Dimitra Bon, Sirkka Schroeter, Alexander Koch, Eva Herrmann, Stefan Zeuzem, Bernd Kronenberger, Josef Pfeilschifter

**Affiliations:** 1 Pharmazentrum Frankfurt, Institut für Allgemeine Pharmakologie und Toxikologie, Frankfurt am Main, Germany; 2 Goethe University Hospital, Medizinische Klinik 1, Frankfurt am Main, Germany; 3 Pharmazentrum Frankfurt, Institut für klinische Pharmakologie, Goethe University Hospital, Frankfurt am Main, Germany; 4 Goethe University, Department of Medicine, Institute of Biostatistics and Mathematical Modelling, Frankfurt am Main, Germany; Medical University of South Carolina, UNITED STATES

## Abstract

**Background:**

Sphingolipids constitute bioactive molecules with functional implications in liver homeostasis. Particularly, ablation of very long chain ceramides in a knockout mouse model has been shown to cause a severe hepatopathy.

**Methods:**

We aimed to evaluate the serum sphingolipid profile of 244 patients with cirrhosis prospectively followed for a median period of 228±217 days via mass spectrometry.

**Results:**

We thereby observed a significant decrease of long and very long chain ceramides, particularly of C24ceramide, in patients with increasing severity of cirrhosis (p<0.001). Additionally, hydropic decompensation, defined by clinical presentation of ascites formation, was significantly correlated to low C24ceramide levels (p<0.001) while a significant association to hepatic decompensation and poor overall survival was observed for low serum concentrations of C24ceramide (p<0.001) as well. Multivariate analysis further identified low serum C24ceramide to be independently associated to overall survival (standard beta = -0.001, p = 0.022).

**Conclusions:**

In our current analysis serum levels of very long chain ceramides show a significant reciprocal correlation to disease severity and hepatic decompensation and are independently associated with overall survival in patients with cirrhosis. Serum sphingolipid metabolites and particularly C24ceramide may constitute novel molecular targets of disease severity, hepatic decompensation and overall prognosis in cirrhosis and should be further evaluated in basic research studies.

## Introduction

Liver cirrhosis constitutes the most severe stadium of chronic liver injury caused by various pathogenic factors and affects approximately 10% of world’s population [1]. 1.8% of all deaths in Europe are attributed to cirrhosis [2] with similar rates observed in the United States [3]. Due to the frequently asymptomatic clinical presentation, cirrhosis remains often unsuspected until clinical complications become apparent. Progression of cirrhosis is often preventable and since it is linked with increased mortality rates, especially when decompensated [4], early diagnosis and adequate monitoring of the disease are indispensable. The Child-Pugh and Model for End-Stage Liver Disease (MELD) scores are the main clinical tools widely deployed to define short term prognosis of affected patients [5,6] but they do not provide evidence on disease progression and dynamic stage of cirrhosis [7]. Incorporation of novel non-invasive markers of disease progression in the currently clinically used scores would improve the identification of severely ill patients and also enable a more accurate organ allocation in patients with end-stage liver disease.

Ceramide (Cer) is the structural and functional hub of sphingolipid metabolism with pleiotropic effects on cellular and molecular physiology of both cell-autonomous systems as well as complex organisms [8]. Cer is synthesized *de novo* from serine and palmitoyl-CoA through the action of various enzymes via dihydro(dh)Cer, through hydrolysis of sphingomyelin by sphingomyelinases and through degradation of complex glucosphingolipids [9]. Though initially suggested as a mere antiproliferative and pro-apoptotic mediator, the effects of Cer on cell fate have been shown to depend substantially on the subcellular compartment of generation and metabolism [10,11] as well as on the chain length of the fatty acid bound to the sphingosine backbone [12,13]. In this context, Cer affects the pathophysiology of various diseases with recent studies suggesting serum Cer levels as possible biomarkers in insulin resistance [14], diabetes mellitus [15], metabolic syndrome [16], Alzheimer’s disease [17] and acute phase reactions [18]. Furthermore, liver pathophysiology is tightly interconnected with sphingolipid (SL) and Cer metabolism since hepatocellular apoptosis is upregulated by Cer accumulation [19–21] and a hepatic Cer increase is associated with the progression of non-alcoholic fatty liver disease (NAFLD) to steatohepatitis [22]. Additionally, myriocin, an inhibitor of *de novo* Cer synthesis, has been shown to inhibit both hepatitis B virus (HBV) [23] as well as hepatitis C virus (HCV) replication [24]. Moreover, recent evidence from a knockout mouse model of Cer-synthase 2, an enzyme mainly expressed in the liver and the kidney which generates *de novo* very long chain Cer’s (C22-C24), has demonstrated increased rates of hepatocellular apoptosis and a severe hepatopathy in these animals with a significant impairment of liver homeostasis [25,26]. In this context, especially very long chain Cer’s appear as critical regulators of liver pathophysiology.

Recently, we were the first to report, that long (C16-C20) and very long (C24, C24:1) chain Cer’s accumulate in the serum of patients with NAFLD as compared to healthy individuals and patients infected with HCV, whereas particularly C24Cer was significantly diminished in the serum of HCV patients compared both to healthy individuals as well as to NAFLD patients [27]. Moreover, in a further study we were able to show that variations of serum SL’s associate significantly with the stage of liver fibrosis and responsiveness to antiviral therapy in chronic HCV infection [28]. Purpose of the current study was therefore to assess serum concentrations of various SL metabolites, including Cer’s with variable chain lengths and particularly very long chain Cer’s, in a prospective series of patients with liver cirrhosis [29] and evaluate potential correlations of bioactive SL’s to severity of cirrhosis, hepatic decompensation and overall survival.

## Materials and Methods

### Patient selection

In the current study we assessed possible correlations of various serum SL parameters with severity of cirrhosis, rate of hepatic decompensation and overall survival in a prospective series of 244 patients with newly or already diagnosed cirrhosis who were treated as in- or out-patients in our department from March 2009 until June 2011 as previously described [29]. Inclusion criteria for the present study were the clinical presentation of cirrhosis, diagnosed either by liver histopathological examination or by pathognomonic results in ultrasound, computer tomography (CT) or magnetic resonance imaging (MRI), age ≥18 years and sufficient storage of serum samples. Patients were excluded from the present analyses if they had received a solid organ allograft within the last 5 years, or if a malignant disease other than hepatocellular carcinoma, was diagnosed. Patients who were eligible for liver transplantation were accordingly listed for liver transplantation, according to Eurotransplant and German guidelines. Patients were included in the study from the day of written informed consent and were followed-up until death, liver transplantation or last contact as previously described [29]. Patients who underwent liver transplantation were excluded from further analysis from the day of transplantation. All serum samples analyzed in this study were taken at the time of admission of the patient in our in-patient or out-patient department. The study was performed in accordance with the Declaration of Helsinki and was approved by the local ethics committee (Ethik-Komission, University Hospital Frankfurt). All patients had signed a written informed consent prior to study inclusion.

### Statistical analysis

Calculations were made with GraphPad Prism v5.01 for Windows (GraphPad Software, San Diego, CA), BiAS software for Windows (version 10.11, Epsilon-Verlag, Darmstadt, Germany) and the free software R (R Foundation for Statistical Computing, Vienna, Austria; http://www.R-project.org software version 3.0.2). Statistical comparisons were carried out using the non-parametric Kruskal-Wallis test with post-hoc tests as well as Mann Whitney-U or the stratified van Elteren tests to determine differences among patient groups. Bonferroni correction was calculated for adjustment of multiple testing if appropriate. Kolmogoroff-Smirnoff’s delta was used to test for Gaussian distributions. The data in the box plots are expressed as Means ± Standard Error unless otherwise specified. Time-dependent receiver operating characteristic (ROC) curves and area under the curve (AUC) values were calculated by the R-software for the evaluation of the predictive accuracy of C24Cer regarding overall survival. For assessment of independent predictors of survival, a time-dependent multivariate analysis was computed by the free software R. Only patients with complete data for the predicting covariates were included in multivariable analysis. Survival curves were calculated with the Cox regression model provided by BiAS. The primary end point was overall survival. The level of significance is set at α = 0.05 representing the 95% confidence interval (CI). Statistically significant differences are indicated in the corresponding Figs: “*” = p<0.05, “**” = p<0.01, “***” = p<0.001.

### Determination of sphingolipid concentrations by high-performance liquid chromatography tandem mass spectrometry

Quantification of serum SL’s was performed by high-performance liquid chromatography tandem mass spectrometry as previously described [27,28]. For quantitation of sphingolipids, 20 μL serum were extracted with methanol:chloroform:HCl (15:83:2). Afterwards amounts of C16:0Cer, C18:0Cer, C20:0Cer, C24:1Cer, C24:0Cer, C16:0dhCer, C18:0dhCer, C24:0dhCer, C24:1dhCer, and the internal standard C17:0Cer; and sphingosine, sphingosine1-phosphate, sphinganine and sphinganine1-phosphate and the internal standards (sphingosine-D7, sphinganine-D7 and sphingosine1-phosphate-D7) were analyzed by liquid chromatography coupled to tandem mass spectrometry (LC-MS/MS). A Luna C18 column (150 mm x 2 mm ID, 5 μm particle size, 100 Å pore size; Phenomenex, Aschaffenburg, Germany) was used for chromatographic separation. The HPLC mobile phases consisted of water-formic acid (100:0.1, v/v) (A) and acetonitrile-tetrahydrofuran-formic acid (50:50:0.1, v/v/v) (B). For separation, a gradient program was used at a flow rate of 0.3 ml/min. The initial buffer composition 60% (A)/40% (B) was hold for 0.6 min and then in 3.9 min linearly changed to 0% (A)/100% (B) and hold for 6.5 min. Subsequently the composition was linearly changed within 0.5 min to 60% (A)/40% (B) and then held for another 4.5 min. The running time for every sample (injection volume: 15 μl for Cer and dhCer determination and 10 μl for the other sphingolipids) was 16 min. MS/MS analyses were performed on a API4000 (triple quadrupole mass spectrometer) equipped with an APCI (Atmospheric Pressure Chemical Ionization) ion source (AB Sciex, Darmstadt, Germany) for Cer and dhCer determination, and with an ESI (Electrospray Ionization) ion source for sphingosine, sphinganine and their 1-phosphate derivatives determination. The analysis was done in Multiple Reaction Monitoring (MRM) mode. For every analyte two transitions were recorded: one for quantification and another one for qualification, to exclude false positive results, with a dwell time of 50 ms. For analysis and quantification the Analyst Software 1.5 (AB Sciex, Darmstadt, Germany) was used and the peak area of the analyte was corrected by the peak area of the internal standard. Linearity of the calibration curve was proven for C16:0Cer, C24:0Cer, C16:0dhCer, C24:1dhCer, C24:0dhCer from 0.6 to 1.000 ng/ml, for C18:0Cer from 0.18 to 300 ng/ml, for C20:0Cer, C24:1Cer from 0.24 to 400 ng/ml) and for C18:0dhCer from 0.3 to 500 ng/ml. For sphingosine, sphinganine and their phosphate derivatives the calibration curve ranged from 0.15 to 250 ng/ml. The coefficient of correlation was at least 0.99. Variations in accuracy were less than 15% over the whole range of calibration. All serum samples were stored at -80°C until assayed.

## Results

### Patient characteristics

The prospective study included 244 patients with cirrhosis of variable etiology followed for a median period of 228±217 days. Hepatic decompensation at inclusion time point was defined as the sole or simultaneous occurrence of ascites, spontaneous bacterial peritonitis (SBP), hepatic encephalopathy (HE), hepatorenal syndrome (HRS) and variceal bleeding and was observed in 73.7% of all patients. A small group of 29 (11.9%) patients was diagnosed with hepatocellular carcinoma. Overall 50 (20.4%) patients died within the study duration. Biochemical, clinical and demographic characteristics of the patients are shown in Table 1.

**Table 1:** Baseline patient characteristics.

Patient demographic, biochemical and clinical data	Median (Range)
Age (years)	57 (25–84)
Sex (female/male) (n, [%])	86 [35,2%] / 158 [64,8%]
ALT (IU/l)	31 (2–1594)
AST (IU/l)	52 (15–2823)
GGT (IU/l)	98.5 (14–1178)
AP (IU/l)	117 (31–688)
LDH (IU/l)	213 (104–1081)
Bilirubin (mg/dl)	2 (0.2–51)
Total serum protein (g/dl)	6.45 (4.1–9.1)
Albumin (g/dl)	3.2 (1.6–5.2)
Creatinine (mg/dl)	0.99 (0.38–5.02)
Sodium (mmol/l)	139 (111–150)
CRP (mg/dl)	1.2 (0–17)
Hemoglobin (g/dl)	10.5 (6.5–16.7)
Platelets (/nl)	100 (17–1507)
INR	1.39 (0.85–4.2)
*Etiology of liver cirrhosis*
Alcohol abuse n, [%]	141 [49.6]
Hepatitis C infection n, [%]	65 [26.6]
Hepatitis B infection n, [%]	30 [12.3]
Non-alcoholic steatohepatitis n, [%]	5 [2.0]
Hereditary hemochromatosis n, [%]	5 [2.0]
Cryptogenic cirrhosis n, [%]	24 [9.8]
Primary sclerosing cholangitis n, [%]	16 [6.6]
Primary biliary cirrhosis n, [%]	3 [1.2]
Autoimmune hepatitis (n, [%])	10 [4.1]
*Severity of liver cirrhosis / HCC occurrence*
MELD-score, median (range)	15 (6–40)
Child-Pugh-stage n, [%]	
A	50 [20.5]
B	121 [49.6]
C	73 [29.9]
Occurrence of HCC n, [%]	29 [11.9%]

*Abbreviations*: ALT: alanine aminotransferase, AST: aspartate aminotransferase, gGT: gamma-glutamyl-transferase, AP: alkaline phosphatase, LDH: lactate dehydrogenase, CRP: c-reactive protein, INR: international normalized ratio, MELD: model of end-liver disease, HCC: hepatocellular carcinoma.

Missing data: *LDH levels were missing in 47 patients*, *Total serum protein levels were missing in 52 patients*, *Albumin levels were missing in 2 patients*, *CRP levels were missing in 4 patients*, *Child score was missing in 3 patients*.

### Low serum long and very long chain (dh-)ceramides associate with severity of liver cirrhosis

**Fig 1:**
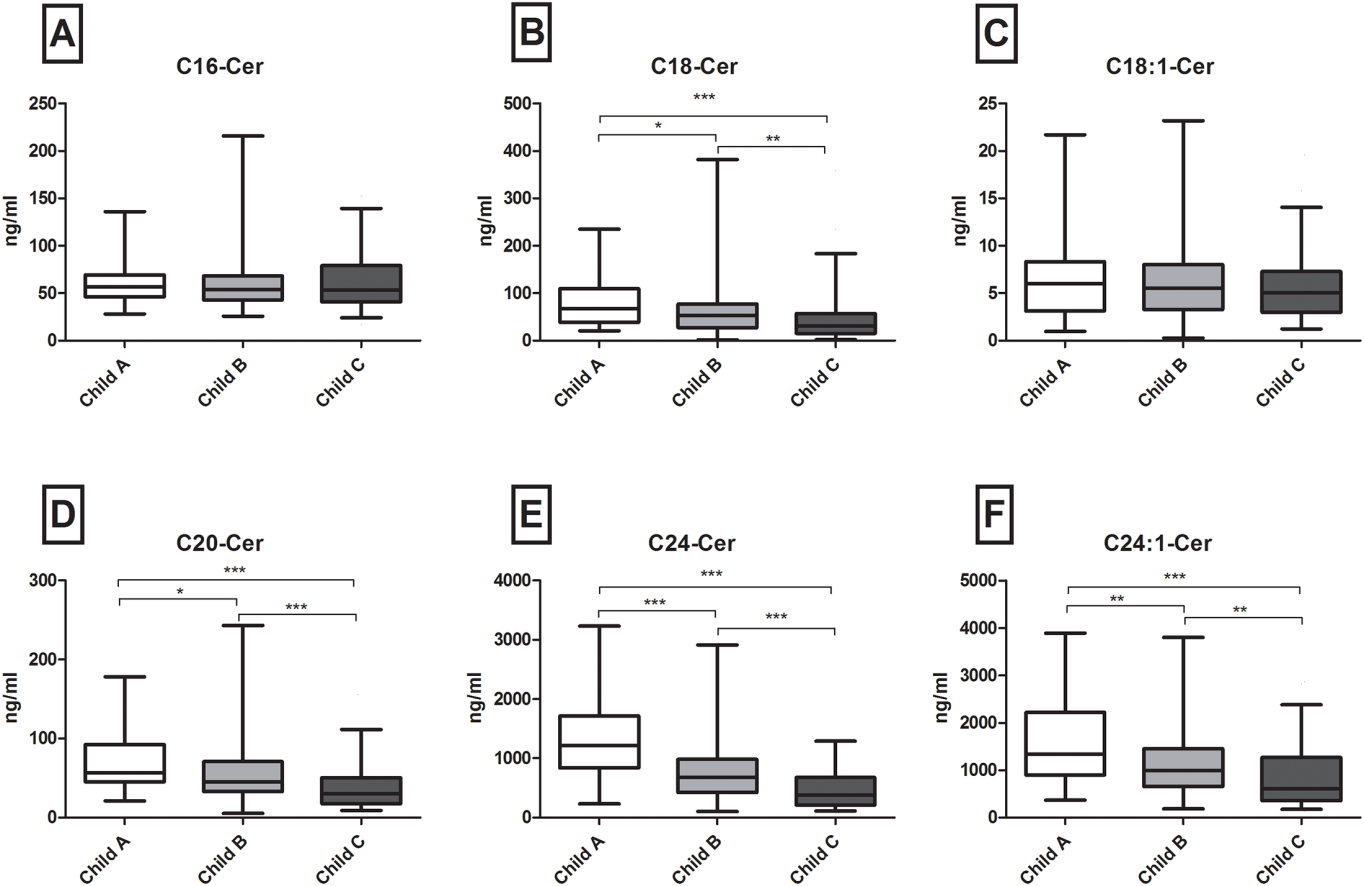
Severity of cirrhosis associates with a decrease of serum Cer levels. C18, C20, C24 and C24:1Cer are significantly decreased with progression of cirrhosis, defined by the Child-Pugh score (p<0.001 in Child A versus Child C cirrhosis respectively). No differences in levels of C16 and C18:1Cer are identified, whereas the most significant decrease is observed in serum C24Cer levels (p<0.001 for all comparisons, 2E). Pairwise comparisons were used as post hoc analysis for significant overall differences. Cer: ceramide.

**Fig 2:**
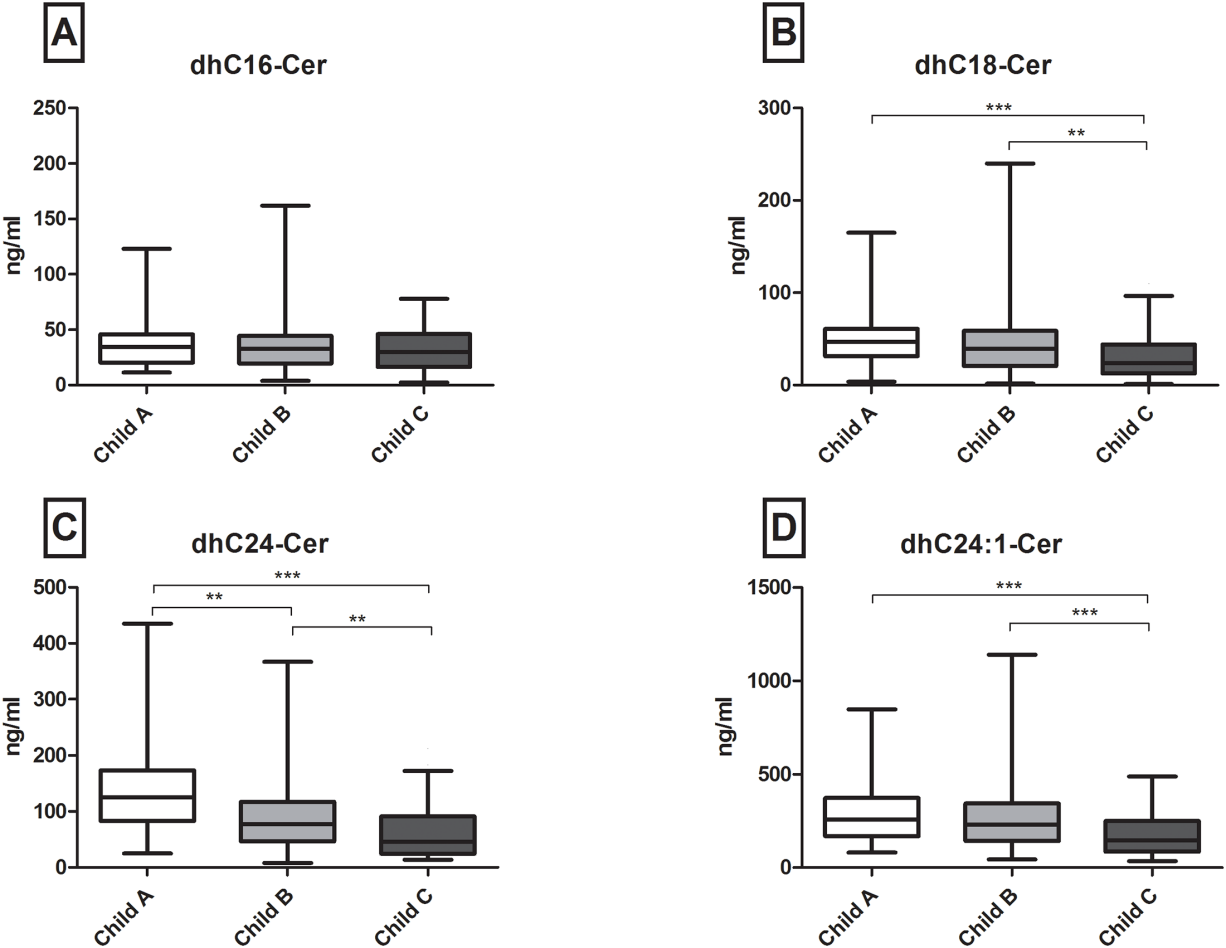
Severity of cirrhosis associates with a decrease of serum dhCer levels. Patients with Child C cirrhosis have significantly lower serum dhCer concentrations as compared to Child A (p<0.001 for dhC18-, dhC24- and dhC24:1-Cer) and Child B cirrhosis (p<0.01 for dhC18- and dhC24-Cer and p<0.001 for dhC24:1-Cer). Only dhC24Cer levels show a significant difference between Child A and Child B cirrhosis (p<0.01). Pairwise comparisons were used as post hoc analysis for significant overall differences. dh: dihydro, Cer: ceramide.

Long chain (C16-C20) and very long chain ceramides (C24, C24:1) are the most abundant SL metabolites in the serum of healthy individuals [30] as well as of patients with chronic liver disease [27]. Similarly, in the present cirrhosis study C24Cer and its unsaturated derivative C24:1Cer showed the highest serum concentrations as compared to C16Cer, C18Cer, C18:1Cer and C20Cer (mean levels of 661 and 992 ng/ml versus 54, 47.1, 5.4, and 43.6 ng/ml respectively). Patients with Child-A cirrhosis showed significantly higher concentrations of long and very long chain Cer’s as compared to patients with Child-C cirrhosis (p<0.001 for C18, C20 and C24, C24:1Cer respectively) (Fig 1). Comparisons between Child-B cirrhosis and Child-A or Child-C cirrhosis revealed marked differences as well (Fig 1), with C24Cer levels showing the highest significance among all Cer species analyzed (p<0.001 for Child-B versus -A and -C, respectively) (Fig 1E). Similar differences were observed in the synthetic precursors of Cer’s, dhCer’s, with dhC24Cer showing the most prominent decrease among all dhCer species assessed (p<0.001 for Child A versus C and p <0.01 for A versus B and B versus C, respectively) (Fig 2). However, no significant associations between serum levels of (dh)C16Cer (Figs 1A and 2A), sphingosine, S1P, dhS1P (Fig A in S1 File) and severity of cirrhosis were observed.

### Serum C24Cer is particularly decreased in HCV cirrhosis

In own previous studies we identified C24Cer being significantly decreased in chronic HCV infection as compared to healthy individuals and patients with NAFLD [27] as well as in HCV patients with severe liver fibrosis [28]. Similarly, in patients with HCV-induced liver cirrhosis serum C24Cer was significantly decreased as compared to cirrhosis induced by other chronic hepatopathies in spite of stratification according to the Child score in order to exclude biased results due to variable severity of cirrhosis (p<0.001, Fig B in S1 File). No significant variations of serum C24Cer levels were observed in patients with cirrhosis caused by chronic alcohol abuse or chronic HBV infection (Fig B in S1 File).

### Low serum Cer concentrations correlate with higher rates of hepatic decompensation

**Fig 3:**
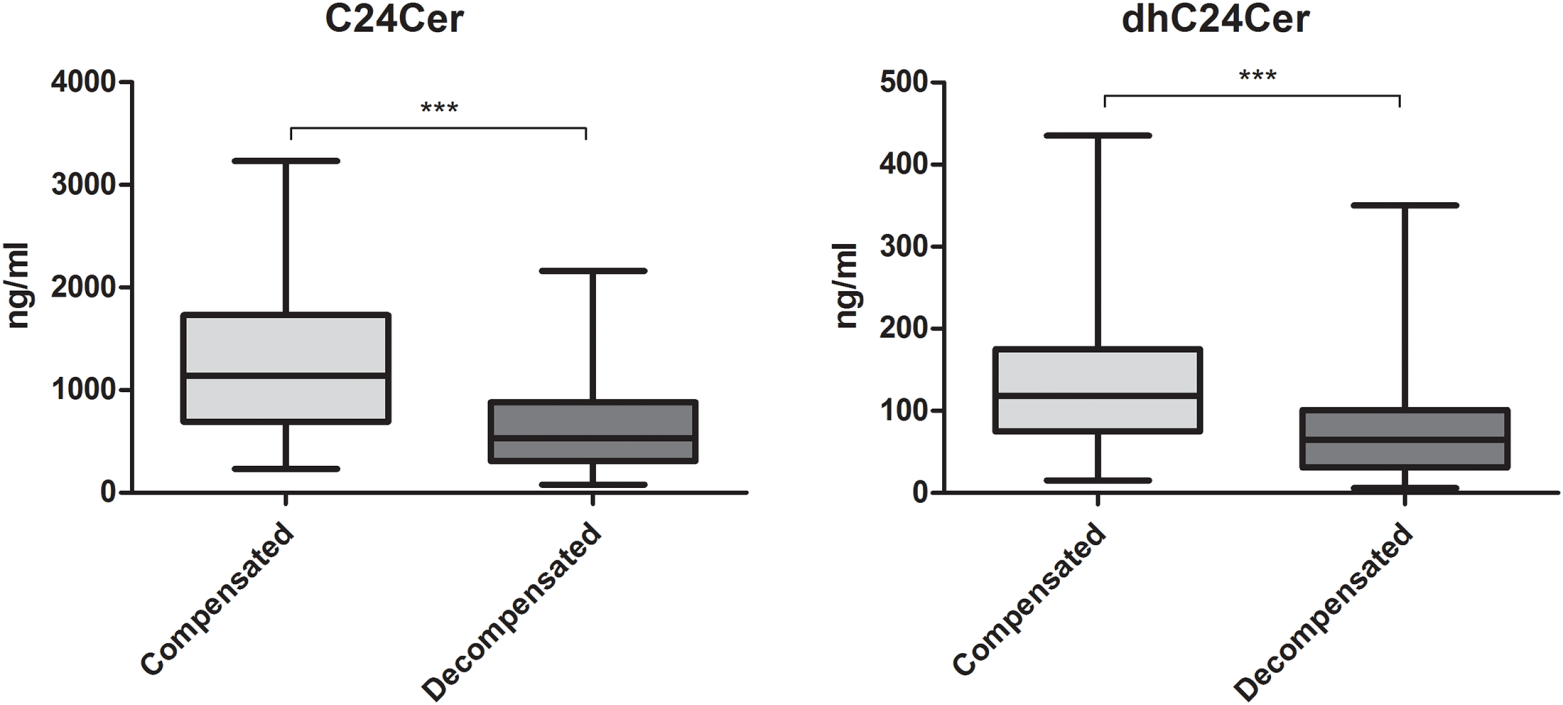
Serum C24Cer and dhC24Cer levels are decreased in decompensated cirrhosis. Serum concentrations of C24Cer and dhC24Cer show a considerable decline in patients with decompensated cirrhosis as compared to patients with compensated cirrhosis (p<0.001). Cer: ceramide.

Decreased serum concentrations of long and very long chain Cer’s associated with the presentation of major clinical complications defining hepatic decompensation such as ascites, SBP, HE and HRS (Table 2). Particularly low serum levels of (dh)Cer’s correlated with a high occurrence of ascites, SBP and HRS in the patients of our study (Table 2). Conversely, the occurrence of HE, apart from a weak significant association to dhC18Cer concentrations (p<0.05), did not correlate to serum Cer (Table 2), while variceal bleeding did not correlate to any of the serum SL parameters assessed. Interestingly, rates of hydropic decompensation, defined by clinical diagnosis of ascites formation, were associated only with serum concentrations of C24Cer and dhC24Cer (p<0.001 and p<0.01 respectively) (Table 2). When the association between overall rate of hepatic decompensation, defined by the occurrence of one or more of the above mentioned events (ascites, SBP, HE, HRS, variceal bleeding), and serum (dh)C24Cer was assessed, we observed a significant decrease of (dh)C24Cer in patients with decompensated disease as compared to patients with compensated cirrhosis (p<0.001, Fig 3).

**Table 2:** Serum sphingolipid concentrations associate with the occurrence of hepatic decompensation in patients with liver cirrhosis. Statistically significant results using Bonferroni adjustment for multiple testing are shown in bold.

	Ascites (median yes/no)	SBP (median yes/no)	HE (median yes/no)	HRS (median yes/no)	Variceal bleeding (median yes/no)
*C16-Cer* (ng/ml)	54.1/53.5	50.9/54.3	55.1/54	47.1/54.9	54.6/54.0
*C18-Cer* (ng/ml)	47/51.3	33.2/50.5	34.4/52	34.6/51.2	44.1/49.5
*C18*:*1-Cer* (ng/ml)	5.3/5.7	4.01/5.57	4.01/5.49	4.96/5.62	5.23/5.48
*C20-Cer* (ng/ml)	43.5/48.4	**29.6/46.6** *	33.7/46.9	**30.5/48.4** ^**^	40.2/43.8
*C24-Cer* (ng/ml)	**572/853** ^***^	**218/702** ^***^	430/702	**305/739** ^***^	580.5/687
*C24*:*1-Cer* (ng/ml)	924/1220	**417/1040** ^**^	756/1040	**522/1100** ^***^	786.5/997
*dhC16-Cer* (ng/ml)	31.2/29.7	23.8/31.3	24.6/31.2	**22.1/32.9** *	33.15/30.6
*dhC18-Cer* (ng/ml)	37.8/37.3	30.7/38.6	**24.2/39.4** *	**24.2/39.1** *	38.4/37.5
*dhC24-Cer* (ng/ml)	**66.1/98.3** ^**^	**24.4/80.7** ^**^	55/81	**28.3/85.2** ^***^	66.15/77.15
*dhC24*:*1-Cer* (ng/ml)	199/233	**92.2/219** ^**^	145/219	**93.8/229** ^***^	164.5/206
*Sph* (ng/ml)	3.9/3.6	3.5/3.9	4.37/3.85	4.04/3.88	5.66/3.72
*S1P* (ng/ml)	112/118	109/113	112/115	123/113	123/112
*dhS1P* (ng/ml)	23/25.4	21.9/23.9	23.9/23.8	24.6/23.6	24.8/23.65

“*” = *P* <0.05,

“**” = *P* <0.01,

“***” = *P* <0.001.

*Abbreviations*: SBP: spontaneous bacterial peritonitis, HE: hepatic encephalopathy, HRS: hepatorenal syndrome, Cer: ceramide, dh: dihydro, Sph: sphingosine, S1P: sphingosine1-phosphate

Missing data: *C18Cer levels were missing in 5 patients out of 244 patient samples*, *dhC16-Cer levels were missing in 1 patient out of 244 patient samples*, *dhC18-Cer levels were missing in 1 patient out of 244 patient samples*, *Sph levels were missing in 17 patients out of 244 patient samples*.

### C24Cer is an independent predictor of survival in cirrhosis

MELD and Child-Pugh score, constitute the “gold standards” to predict mortality rates in patients with cirrhosis [5,6]. Since the decrease of serum C24Cer and dhC24Cer levels associated significantly with the severity of cirrhosis (Figs 1 and 2) as well as with the incidence of hepatic decompensation (Table 2, Fig 3), a correlation of serum C24Cer and dhC24Cer levels with short-term mortality appears plausible. Thus, in order to assess the predictive potential of C24Cer in the estimation of overall survival we conducted a multivariate analysis including the variables sex, age, MELD score, HCV-infection, alcohol abuse as well as C24Cer in patients with cirrhosis (Table 3). In order to exclude collinearity between the MELD and the Child score we conducted two separate multivariable analyses, each of them including only the MELD or the Child score in each case (Table 3). According to our data, MELD score (standard β = 0.109, p<0.001), age (standard β = 0.032, p = 0.035), alcohol abuse (standard β = -0.655, p = 0.045) and C24Cer (standard β = -0.001, p = 0.022) were independent predictors of overall survival in patients with cirrhosis (Table 3). When Child score replaced the MELD score in the multivariable analysis C24Cer remained as an independent predictor of overall survival (Table 3).

**Table 3:** Multivariate analysis of variables associated with overall survival in patients with liver cirrhosis.

**Variables**	***Standard Beta***	***P value***
MELD score (continuous)	0.109	<0.001
Sex (male/female)	0.006	0.8
Age (years, continuous)	0.032	0.035
HCV infection (yes/no)	0.119	0.7
Alcohol abuse (yes/no)	-0.655	0.045
C24Cer (ng/ml, continuous)	-0.001	0.022
Variables	***Standard Beta***	***P value***
Child B stage	0.804	0.2
Child C stage	1.409	0.06
Sex (male/female)	0.735	0.032
Age (years, continuous)	0.028	0.06
HCV infection (yes/no)	0.270	0.4
Alcohol abuse (yes/no)	-0.578	0.08
C24Cer (ng/ml, continuous)	-0.001	0.005

Only patients with complete data for the remaining covariates were included in multivariate analyses. Significant values are shown in italic fonts. *Abbreviations*: MELD: model of end stage liver disease, HCV: hepatitis C virus, Cer: ceramide.

Missing data: *In the multivariate analysis including the MELD score data for presented variables were available for 216 out of 244 patients. In the multivariate analysis including the Child score data for the presented variables were available for 213 out of 244 patients*.

### The predictive accuracy of the MELD score is upgraded by inclusion of C24Cer regarding overall survival in patients with decompensated cirrhosis

**Fig 4:**
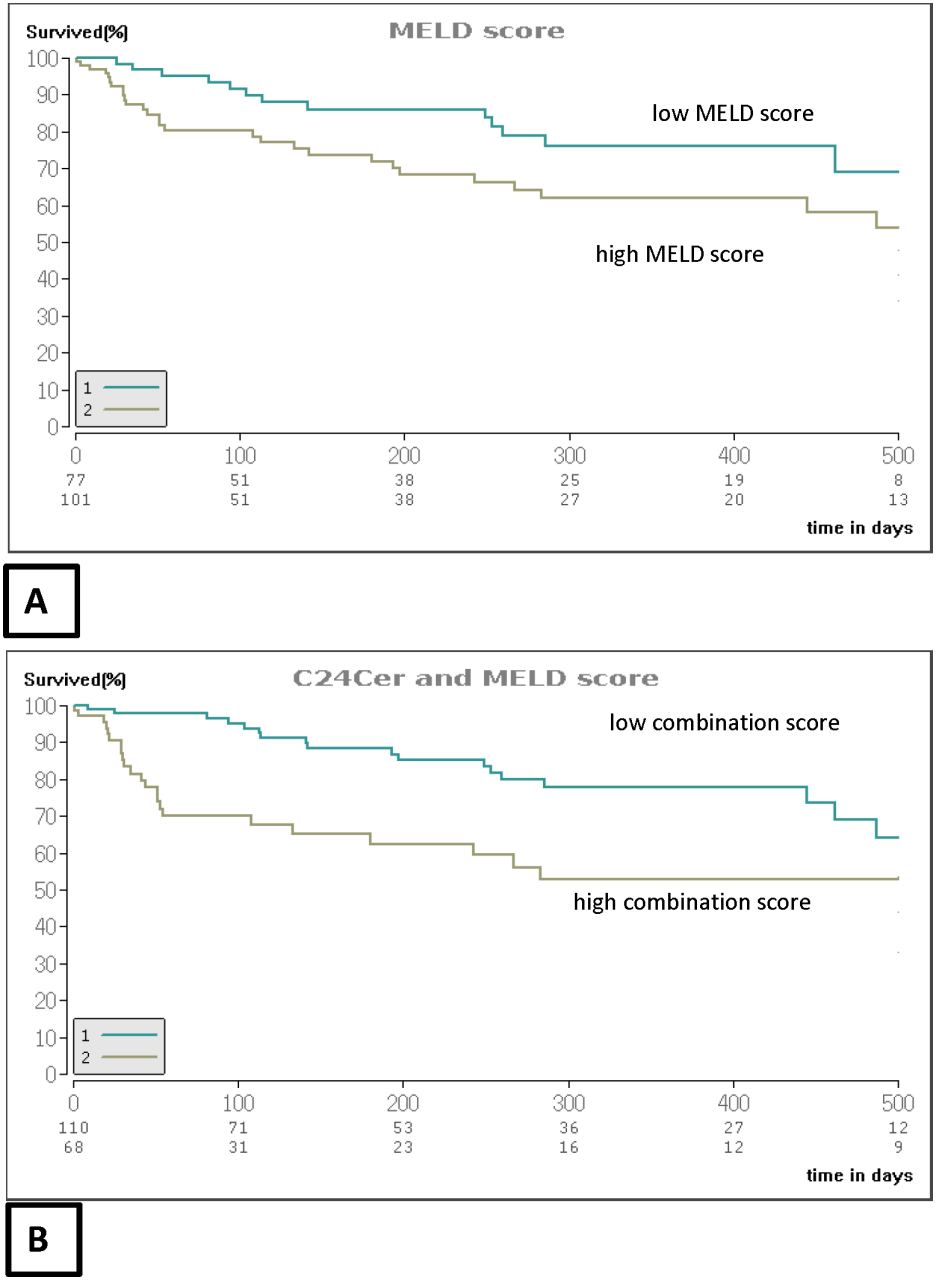
Overall survival in patients with decompensated cirrhosis after stratification to C24Cer and MELD. Cumulative survival is significantly diminished in patients with low C24Cer (≤ 391 ng/ml) and high MELD score (≥15) (p<0.001). As a reference the cumulative survival of patients according to the MELD score is illustrated as well (p<0.001). Cumulative survival was calculated with the univariate Cox regression model. MELD: model of end stage liver disease, Cer: ceramide.

When overall survival was evaluated in patients with low C24Cer (≤397 ng/ml; cut-off identified by time-dependent survival ROC analysis), a significant decrease of cumulative survival was identified as compared to patients with higher C24Cer values (p<0.001) comparable to the cumulative survival observed according to high (≥15) and low (<15) MELD values (p<0.001, Fig 4). Though, since low C24Cer correlated significantly with high MELD values (rho = -0.391, p<0.001, Fig C in S1 File) we compared the predictive accuracy of both parameters regarding overall survival in separate ROC survival analyses. Thereby, similar AUC values were observed for C24Cer and the MELD score (0.704 and 0.715 respectively, p<0.001 for both parameters, Fig D in S1 File). Thus, we further evaluated the diagnostic performance of an updated MELD score through inclusion of C24Cer by using the formula: “MELD+0.02*(600-C24Cer)” (Fig 4). Patients with a low combination score showed higher survival rates than patients with a high combination score (Fig 4).

## Discussion

We have previously observed a deregulation of bioactive SL’s with major impact on hepatic pathophysiology, particularly Cer concentrations, in the serum of patients with chronic liver disease as compared to healthy individuals [27] as well as in HCV patients with severe liver fibrosis [28]. Therefore, aim of the present study was to assess the serum SL profile in cirrhosis as well as to evaluate the predictive potential regarding disease progression, hepatic decompensation and overall survival in cirrhosis.

Our current analysis focused on the serum concentrations of very long chain Cer’s and long chain Cer’s in patients with cirrhosis since on the one hand many of the enzymes producing very long chain Cer’s, as for instance Cer synthase 2, are mainly expressed in the liver [12] and on the other hand variations of very long chain Cer’s have been shown to mediate critical molecular changes in liver pathophysiology leading to a severe hepatopathy [25,26]. We observed a significant decrease in the serum levels of C24Cer in cirrhotic patients as compared to healthy individuals in previously published data both from our group [27] as well as from other groups [30]. In particular, the mean concentration of C24Cer in 241 cirrhotic patients of the current study was 487.9 ± 375 ng/ml compared to a mean level of 4225.3 ± 1965.4 ng/ml of 71 healthy donors assessed in our clinic [27] and similar high levels of >4.0 μM in 10 healthy individuals as previously published by Hammad et al. [30]. Interestingly, in our current study the mean serum levels of C24Cer, which represents the most abundant Cer in serum, were lower than the levels of C24:1Cer (487.9 ± 375 and 851.5 ± 675 ng/ml respectively). Thus, a marked decrease in serum levels of C24Cer may constitute a novel marker of cirrhosis, a hypothesis that should be certainly verified by further studies.

Further, our findings show that low serum C24Cer is significantly associated with the rate of hepatic decompensation (Fig 3) and overall survival (Table 3). Since HCV infection showed both in previous own observations [27,28] as well as in the current study a significant decrease of serum C24Cer in respective patients we included HCV infection in the multivariable prediction model of overall survival in order to exclude possible bias in the predictive potential of C24Cer (Fig B in S1 File). C24Cer remained associated with overall survival independently of HCV infection (Table 3). Beside the observed significant inverse correlation of serum C24Cer levels to severity of cirrhosis (Fig 1) and occurrence of hepatic complications (Table 2), C24Cer correlated significantly with the MELD score (Fig C in S1 File), while in a further time-dependent ROC analysis of overall survival the AUC of C24Cer appeared similar to that of the MELD score (Fig D in S1 File), thus indicating that serum C24Cer associates significantly with overall mortality in the current prospective series of patients with cirrhosis. When only patients with decompensated cirrhosis were included in the analysis, low C24Cer associated with survival independently of the Child score, whereas the MELD score prevailed over C24Cer in the estimation of overall survival (Table A in S1 File). We further evaluated the predictive accuracy of an updated prediction score by combining C24Cer with the MELD score. Combination of MELD and C24Cer appeared superior in predicting survival for this time period as compared to the MELD score alone (Fig 4). In this regard, the present study shows to our knowledge for the first time that low serum C24Cer associates independently with poor survival in patients with cirrhosis. In this context, a recently published study on the plasma sphingolipid profile of patients with type 1 diabetes revealed that low levels of very long chain Cer’s were associated with the development of macroalbuminuria in these patients [31]. Thus, very long chain Cer’s appear to play a protective role in hepatic and renal homeostasis and their significant decrease may be predictive of organ failure. The underlying mechanisms should be further evaluated in basic research studies.

Interestingly, we additionally observed that clinical presentation of ascites showed a significant inverse correlation solely to the levels of C24Cer and dhC24Cer and not to other (dh)Cer species (Table 2). The occurrence of SBP and HRS, both conditions clinically closely related to the presence of ascites, also correlated to significant variations in the serum levels of C24Cer and dhC24Cer (Table 2). From a mechanistic point of view previous studies have demonstrated, that the high hydrophobic properties of very long chain Cer’s are substantially regulating cell membrane fluidity and consequently also water permeability of the human skin [32,33]. Additionally, liver microsome membranes isolated from a knockout mouse model of Cer synthase 2, with a consecutive lack in very long chain Cer’s, were markedly less fluid [26], a factor critically affecting membrane permeability and thus possibly also the formation and/or defective reabsorption of ascites. Certainly further basic research and translational studies are required in order to decipher the role of very long chain Cer’s in the pathophysiology of ascites formation.

However, the highly interesting novel associations identified by our analysis raise the question about the underlying mechanism of C24Cer decrease in cirrhosis and the biological significance of this phenomenon. Numerous studies have attributed critical second messenger functions to Cer including the orchestration of cellular responses upon various stimuli and the induction of apoptosis, senescence and autophagy [34,35]. Yet, as mentioned before, depending on the chain length and saturation of the fatty acid [12], as well as on the tissue or cell specific compartment of Cer generation and metabolism [11], particularly very long chain Cer’s (≥C24) have been shown to promote proliferation in vitro [36,37] contrary to the pro-apoptotic effects of long chain Cer’s, especially C16Cer, in various cell types [38,39]. In hepatic pathophysiology, massive apoptosis of hepatocytes induces an activation of hepatic stellate cells which produce collagen, promote fibrosis and ultimately cirrhosis of the liver [40,41]. Accordingly, inhibiting hepatic apoptosis is considered as an important strategy to prevent cirrhosis [42,43] while a recent study identified an association of apoptotic markers to increased severity of infections and higher mortality in these patients [44]. Moreover, ablation of Cer synthase 2 in respective knockout mice with a consecutive depletion of very long chain Cer’s has been shown to induce chronic oxidative stress, in part responsible for the progressive and severe hepatopathy observed in these mice [45]. In line with these observations, our current study suggests that the identified decrease in serum C24Cer probably shifts the balance between proliferation and apoptosis in favor of a pro-apoptotic metabolic state, also exposed to chronic oxidative stress, and thus deteriorate clinical decompensation and mortality in cirrhosis. Definitely, further studies are required, in order to address the pathogenic significance and role of very long Cer’s in cirrhosis.

Although offering promising results our study had some limitations. As a clinical association study it cannot proof a causal relationship between SL metabolism and cirrhosis. Since serum Cer’s are bound to lipoproteins such as low density lipoprotein (LDL), high density lipoprotein (HDL) and very low density lipoprotein (VLDL) it cannot be excluded that the observed differences are due to changes of the mentioned lipoproteins in patients with cirrhosis. Unfortunately, serum levels of these lipoproteins were not available in our patients. Most of the included patients showed a hepatic decompensation at time of inclusion and follow-up data regarding clinical recompensation or anew decompensation were unfortunately not available. Despite our effort to provide a comprehensive multivariable analysis regarding prediction of overall survival, data on prevalence of diabetes mellitus, metabolic syndrome and insulin resistance were lacking in our analysis. Nevertheless, our observations offer a suggestive functional link between SL’s and cirrhosis and thus a significant advance over previous studies since they demonstrate significant variations in serum SL’s according to different severity, decompensation and overall survival of cirrhosis.

In conclusion, our study identified, to our knowledge for the first time, very long chain Cer’s and particularly C24Cer to be associated with hepatic decompensation and to correlate independently with overall survival of end-stage liver disease. Particularly, an updated MELD score by inclusion of C24Cer, showed an increased predictive accuracy regarding overall survival in patients with decompensated cirrhosis. Certainly further studies are needed in order to elucidate the underlying mechanism as well as the role of serum SL’s and particularly C24Cer in the non-invasive mortality prediction in patients with cirrhosis.

## Supporting Information

S1 File Supplementary Materials. (DOCX)

## List of abbreviations

MELD: model for end stage liver disease
Cer: ceramide
SL: sphingolipid
NAFLD: non-alcoholic fatty liver disease
HBV: hepatitis B virus
HCV: hepatitis C virus
dh: dihydro
CT: computer tomography
MRI: magnetic resonance imaging
SBP: spontaneous bacterial peritonitis
HE: hepatic encephalopathy
HRS: hepatorenal syndrome
PSC: primary sclerosing cholangitis
ESI: electrospray ion source
MRM: multiple reaction monitoring
ROC: receiver operating characteristic
AUC: area under the curve
HR: hazard ratio
CI: confidence interval
CRP: C reactive protein
LDL: low density lipoprotein
HDL: high density lipoprotein
VLDL: very low density lipoprotein
