# Supplementary material for: Serum Sphingolipid Variations Associate with Hepatic Decompensation and Survival in Patients with Cirrhosis
Source: PLoS One. 2015 Sep 18;10(9):e0138130. doi: 10.1371/journal.pone.0138130 (PMC4575185; doi:10.1371/journal.pone.0138130)
Supplement: S1 File — (DOCX) [file pone.0138130.s001.docx]

***SUPPLEMENTARY MATERIAL***

Serum sphingolipid variations associate with hepatic decompensation and survival in patients with cirrhosis

Georgios Grammatikos^1,2^, Nerea Ferreiròs^3^, Oliver Waidmann^2^, Dimitra Bon^4^, Sirkka Schroeter^1,2^,Alexander Koch^1^, Eva Herrmann^4^, Stefan Zeuzem^2^, Bernd Kronenberger^2^ and Josef Pfeilschifter^1^

1. Pharmazentrum Frankfurt, Institut für Allgemeine Pharmakologie und Toxikologie, Frankfurt am Main, 2. Goethe University Hospital, Medizinische Klinik 1, Frankfurt am Main, 3. Pharmazentrum Frankfurt, Institut für klinische Pharmakologie, Goethe University Hospital, Frankfurt am Main, 4. Goethe University, Department of Medicine, Institute of Biostatistics and Mathematical Modelling, Frankfurt am Main, Germany.

**Supplementary Table**

**Table A** Multivariate analysis of variables associated with overall survival in decompensated patients with liver cirrhosis

| **Variables** | ***Standard Beta*** | ***P value*** |
| --- | --- | --- |
| MELD score (continuous) | *0.101* | *<0.001* |
| Sex (male/female) | *0.879* | *0.011* |
| Age (years, continuous) | 0.025 | 0.1 |
| HCV infection (yes/no) | 0.289 | 0.4 |
| Alcohol abuse (yes/no) | -0.524 | 0.1 |
| C24Cer (ng/ml, continuous) | -0.0009 | 0.09 |
| **Variables** | ***Standard Beta*** | ***P value*** |
| Child B stage | 16.11 | 0.9 |
| Child C stage | 16.77 | 0.9 |
| Sex (male/female) | 0.714 | 0.051 |
| Age (years, continuous) | 0.025 | 0.1 |
| HCV infection (yes/no) | 0.408 | 0.2 |
| Alcohol abuse (yes/no) | -0.442 | 0.3 |
| C24Cer (ng/ml, continuous) | *-0.130* | *0.002* |

Only patients with complete data for the remaining covariates were included in multivariate analyses. Significant values are shown in italic fonts. *Abbreviations:* MELD: model of end stage liver disease, HCV: hepatitis C virus, Cer: ceramide. *Missing data: In the multivariate analysis including the MELD score data for presented variables were available for 158 out of 180 patients. In the multivariate analysis including the Child score data for the presented variables were available for 156 out of 180 patients.*

**Supplementary Figures**

**Fig A Serum bioactive SL and severity of liver cirrhosis**

**
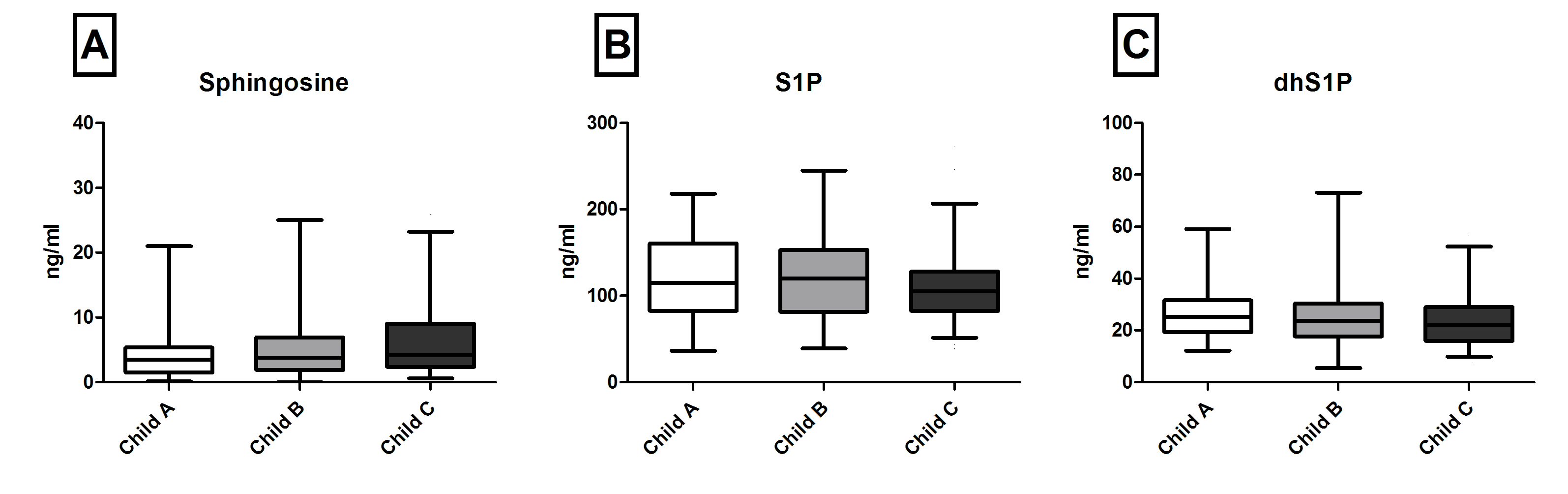
**

**Fig B Etiology of liver cirrhosis and serum C24Cer stratified by Child score**

**
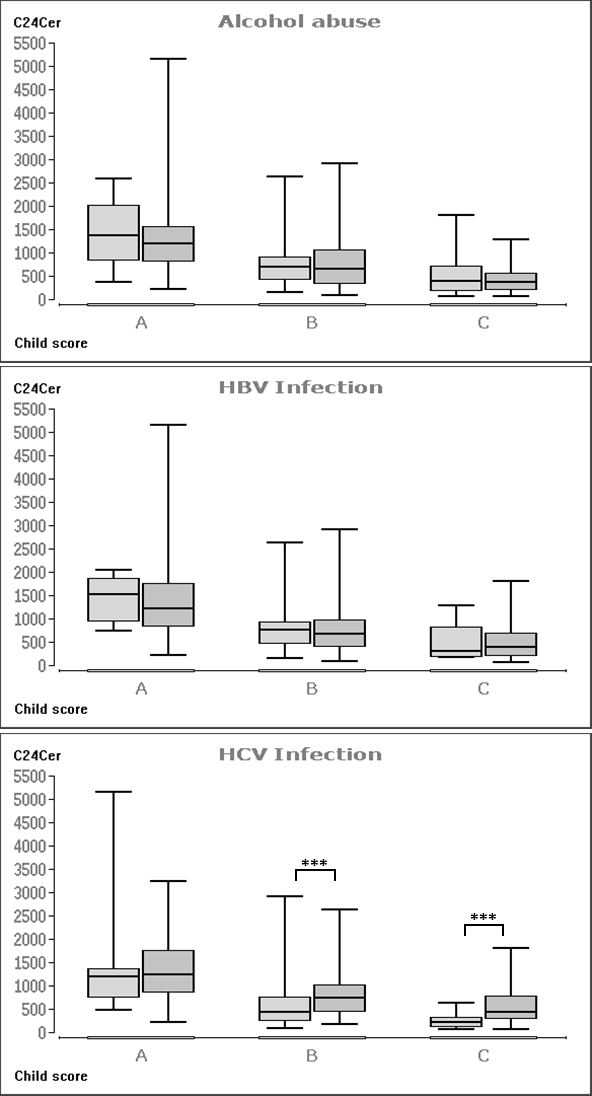
**

**Fig C Correlation of C24Cer and MELD score**

**
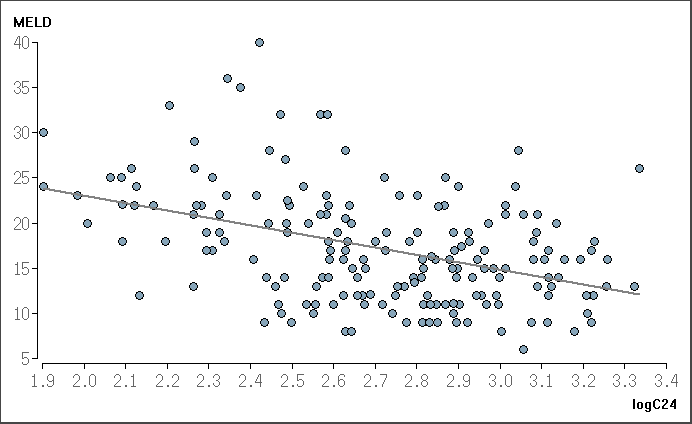
**

**Fig D Survival ROC analysis for C24Cer and MELD score**

**
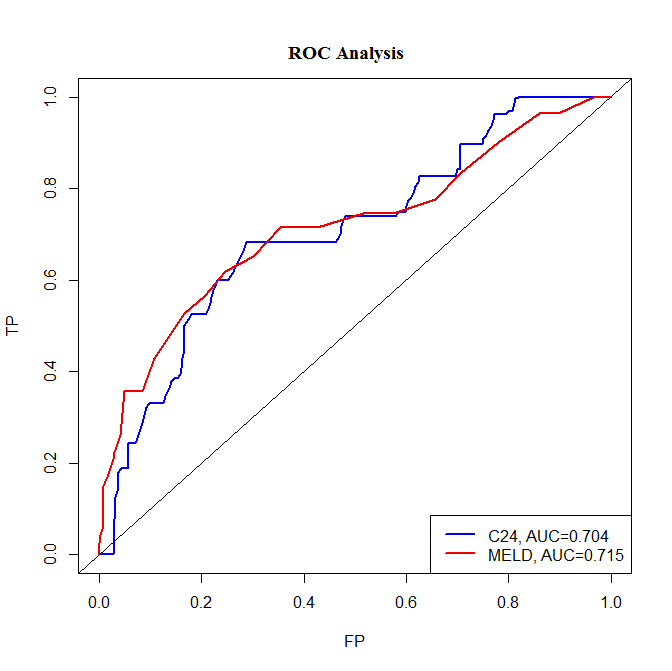
**

**Supplementary figure legends**

**S1 Figure**

No significant variations are observed in the levels of sphingosine, S1P and dhS1P among different stages of liver cirrhosis. S1P: sphingosine 1-phosphate, dhS1P: dihydro-sphingosine1-phosphate.

**S2 Figure**

Stratified analysis with post-hoc comparisons is shown in case of significance. The observed decrease of C24Cer in HCV infection is independent of the Child score and particularly significant for Child stage B and C (p<0.001). No significant variations in chronic HBV infection and in alcohol consume are identified. Dark grey box plots illustrate absence of alcohol abuse, HBV and HCV infection respectively. HCV: hepatitis C virus, HBV: hepatitis B virus.

**S3** **Figure**

Serum levels of C24Cer show a significant inverse correlation to MELD score (rho= -0.391, p<0.001). Spearman’s rho was calculated via Spearman rank correlations.

**S4 Figure**

Time-dependent survival ROC analysis with the respective AUC values was calculated to evaluate the diagnostic performance of C24Cer as compare to the MELD score. A significant association for both C24Cer and MELD values regarding prediction of overall survival was observed (p<0.001) with similar AUC values (0.704 and 0.715) for both parameters respectively.
